# The Price of Sex: Condom Use and the Determinants of the Price of Sex Among Female Sex Workers in Eastern Zimbabwe

**DOI:** 10.1093/infdis/jiu493

**Published:** 2014-12-01

**Authors:** Jocelyn Elmes, Kundai Nhongo, Helen Ward, Timothy Hallett, Constance Nyamukapa, Peter J. White, Simon Gregson

**Affiliations:** 1 Department of Infectious Disease Epidemiology; 2 MRC Centre for Outbreak Analysis and Modelling; 3 NIHR Health Protection Research Unit in Modelling Methodology, Department of Infectious Disease Epidemiology, School of Public Health, Imperial College London; 4 Modelling and Economics Unit, Centre for Infectious Disease Surveillance and Control, Public Health England, London, United Kingdom; 5 Biomedical Research and Training Institute, Avondale, Zimbabwe

**Keywords:** female sex work, payments, condom use, sub-Saharan Africa

## Abstract

***Background.*** Higher prices for unprotected sex threaten the high levels of condom use that contributed to the decline in Zimbabwe's human immunodeficiency virus (HIV) epidemic. To improve understanding of financial pressures competing against safer sex, we explore factors associated with the price of commercial sex in rural eastern Zimbabwe.

***Methods.*** We collected and analyzed cross-sectional data on 311 women, recruited during October–December 2010, who reported that they received payment for their most-recent or second-most-recent sex acts in the past year. Zero-inflated negative binomial models with robust standard errors clustered on female sex worker (FSW) were used to explore social and behavioral determinants of price.

***Results.*** The median price of sex was $10 (interquartile range [IQR], $5–$20) per night and $10 (IQR, $5–$15) per act. Amounts paid in cash and commodities did not differ significantly. At the most-recent sex act, more-educated FSWs received 30%–74% higher payments. Client requests for condom use significantly predicted protected sex (*P* < .01), but clients paid on average 42.9% more for unprotected sex.

***Conclusions.*** Within a work environment where clients' preferences determine condom use, FSWs effectively use their individual capital to negotiate the terms of condom use. Strengthening FSWs' preferences for protected sex could help maintain high levels of condom use.

Price premiums for unprotected sex with female sex workers (FSWs) could limit the success of behavioral interventions aimed to increase condom use in commercial transactions [1–3]. FSWs have reported financial coercion (ie, clients’ refusal to pay unless sex is unprotected) [2] and willingness not to use condoms for a higher price [1–3]. Behavioral economists have argued that the price of unprotected sex is determined by FSW preferences for protection, client preferences for unprotected sex, and the respective bargaining powers of each group [4]. Targeted interventions may strengthen FSW preferences for condom use, which then require greater premiums to reverse [3]. Conversely, illness or economic shocks at individual, household, or community levels may increase willingness to supply unprotected sex to meet additional expenditures [5, 6].

Since 2000, Zimbabwe has experienced a prolonged economic decline, with hyperinflation, food shortages, and deep poverty [7]. Zimbabwe's human immunodeficiency virus (HIV) epidemic is one of the severest worldwide but has declined partly because of behavior change, including increased condom use between commercial sex partners [8, 9].

During the economic collapse, rural sex work became more diffuse and less professional, and sex was sold for commodities in lieu of cash [10]. However, little is known quantitatively about factors affecting the price of sex. Understanding financial pressures competing against safer sex and the factors that increase commercial payments in FSWs is key to designing interventions that promote condom use or aim to incentivize behaviors, such as cash transfers [11].

We identify potential determinants of price in the FSW population that could confound the relationship between condom use and price, examine the association of condom use with client and FSW preferences for protected sex, and examine differences in the prices of protected and unprotected sex.

## METHODS

### Data Collection

Data came from round 2 of an open-cohort study conducted in 4 neighboring but socioeconomically diverse communities—a small town, a roadside trading center, a commercial forestry estate, and a subsistence farming area—in Manicaland, eastern Zimbabwe [10]. Data collection methods are described in the Supplementary Materials. Women aged ≥18 years who reported ever having had transactional sex were recruited between March 2010 and May 2011, using venue-based and snowball methods. To participants providing informed consent, trained interviewers asked questions face to face about demographic characteristics and sexual behavior. In informal confidential voting interviews designed to reduce social-desirability bias [12], respondents self-completed answers to questions about their 2 most-recent sex partners (none were illiterate). Ethical approval was obtained from the Medical Research Council of Zimbabwe (MRCZ/A/681) and Imperial College Research Ethics Committee (ICREC_9_3_13).

### Outcome and Exposure Variables

We restricted analysis to women who reported in informal confidential voting interviews that at least one of their 2 most-recent sex partners within the past year was commercial.

The primary outcome was the amount in 2010 US dollars (or equivalent value of goods) received for commercial sex. We included so-called zero-payments from clients, to allow for those who pay on credit at the end of an agreed period instead of per encounter [10, 13]. Missing payments were excluded (sensitivity analyses are in the Supplementary Materials).

Condom use during the most-recent sex encounters (an encounter can comprise ≥1 sex act) was measured as the number of protected acts. Since most individuals reported that either all (62.4%) or no acts were protected (30.8%), condom use was coded as a binary variable by subtracting the number of protected acts from the total number of acts reported to represent consistent use of condoms (ie, all acts were protected) versus inconsistent use or nonuse of condom (not all acts were protected).

#### Potential Determinants of the Price of Sex Among FSWs

A literature review identified a number of factors associated with price that could be confounders. Positively associated factors included FSW attractiveness [4], duration in sex work [3], and certain sexual practices (eg, anal and oral sex) [14]. Increasing age [3] and FSW poverty [15] were negatively correlated with price. We used age as a proxy for physical attractiveness [3, 4], occupation and marital status as proxies for FSW wealth (with the assumption that a union implies dual incomes), and dry sex (ie, drying of the vaginal passage to increase pleasure) as an example of premium service. Repeat clients may negotiate lower prices [16], condom use is often lower than with new clients [17]), and client preference for unprotected sex is associated with price [4]. Women in poorer areas may be more willing to accept incentives for unprotected sex [2]. As an indicator of socioeconomic status [18], study area was considered as a group-level factor. The number of areas (4) was considered to be too small for the size of the data set to detect variation between groups in multilevel modeling [19], but we tested spatial clustering with the intraclass cluster coefficient [19, 20].

Preliminary analyses tested differences in potential determinants of price between women reporting one of the last 2 partners as commercial (FSW1s) and women reporting that both were commercial (FSW2s), using χ^2^ analysis (or the Fisher exact test) and the Wilcoxon rank-sum test for continuous variables. In these analyses, the 2 groups were significantly different, so they were subsequently analyzed separately.

#### Client and FSW Preferences for Protected Sex and Association With Condom Use

We used variables that separately denoted client and FSW requests for condoms as a proxy for their preferences. Positive answers indicated a preference for condom use, and we interpreted negative responses as indicating no preference for protected sex. Spearman correlation coefficients, stratified by the request type (ie, client, FSW, and joint), were used for testing the null hypothesis that number of acts and number of protected acts were independent (ie, a measure of consistency of condom use).

#### Expected Payments for Sex

The paired Wilcoxon signed rank test was used to test whether the amount each woman expected to receive (ie, prices) for protected sex differed from that received for unprotected sex, whether the amount received for a full-night sexual encounter differed from that received for a short-duration sexual encounter (usually a single sex act), and whether these expectations were met in FSWs' most-recent commercial encounters.

#### Independent Determinants of the Price of Sex

Separate multivariable regressions were conducted for the 2 FSW groups. Factors significant at a *P* value of <.2 in univariate analyses were included in the multivariable model [21]. Discrete and continuous variables were described by median and interquartile ranges (IQRs). Continuous variables were mean centered; nonlinear relationships were explored by categorizing into quartiles. Age was categorized into 18–24, 25–34, 35–44, and ≥45 years. Final model variables were selected from full models by means of backward stepwise elimination (threshold to remove: *P*<.05), using likelihood ratio tests between nested models and minimizing the Akaike information criterion (AIC) and Bayesian information criterion (BIC) to select between different nonnested models (Wald tests replaced likelihood ratio tests when sandwich estimators were used [20]).

We regressed price on its predictors by using zero-inflated negative binomial (ZINB) models to account for nonnegative, overdispersed distributions with excess zeros [22], allowing inclusion of clients who paid on credit and so may have given zero payment at the most-recent sex encounter—so-called structural zeros [23]. The ZINB model assumes that the data are generated by a mixture of 2 processes. A Bernoulli trial determines with a probability *p* whether a zero count is produced or with a probability [1 − *p*] whether a count (either zero or positive) is generated from a negative binomial distribution. Coefficients from the count part of the model are interpreted as coefficients from a negative binomial (NB) regression; coefficients from the binary part are interpreted as logistic-regression coefficients [22]. For FSW2s, we compared final models with unclustered and robust standard errors clustered on the individual to adjust for the underlying correlation within FSWs.

Model fit was assessed using the AIC, the BIC, and the Vuong test to discriminate between NB and ZINB models [22]. We assessed the sensitivity of the final model to its inputs by exploring misclassification bias and outlier influence [20, 21] (Supplementary Information).

Relationships between predictors and payments in the count part of the ZINB are interpreted as percentage changes in payments for a unit of change in the predictor (derived from the NB coefficients), holding all other variables constant. Association of a particular predictor and zero counts in the binary part of the ZINB is interpreted as the odds of zero payments in excess of that expected under a NB process. Analyses were conducted in Stata/SE 11.2 (StataCorp).

## RESULTS

Of 458 observations in 311 women who reported having a commercial sex partner in the past year, there were 134 and 136 reported outcomes for the most-recent commercial partner of FSW1s and FSW2s, respectively, and 143 observations for the second-most-recent commercial partner of FSW2s.

### Characteristics and Determinants of Price of Sex Among FSWs

Across a range of characteristics, FSW1s were significantly different from FSW2s (Table 1). FSW1s were older, less likely to report sex work as their occupation, started sex work later, had been selling sex for fewer years, were less likely to request condom use, were more likely to have clients paying on credit, and were less likely to self-identify as a FSW in the last year (25.5% vs 63.3%; *P* < .01). Over the 6 months prior to completing the survey, 76.2% of FSW1s regularly sold sex on credit, and 37.8% sold sex per encounter; 74.2% and 76.9% of FSW2s regularly sold sex on credit and per encounter, respectively. The median duration since most-recent commercial sex was 21 days (IQR, 7–100 days) for FSW1s and 7 days (IQR, 2–16 days) for FSW2s.

**Table 1.** Tests for Difference in Sociodemographic and Behavioral Characteristics of Female Sex Workers Reporting 1 Commercial Sex Partner at the Most Recent Sex Act (FSW1) or 2 Commercial Sex Partners at the Most Recent and Second Most Recent Sex Encounters (FSW2) in the Past Year and Univariable Associations With Price

Characteristics	FSW1		FSW2 (Most Recent Commercial Partner)		Test for Difference
	Proportion or Median	Univariable ZINB β Coefficient (95% CI)	Proportion or Median	Univariable ZINB β Coefficient (95% CI)	*P* Value
A: General sociodemographic and behavioral characteristics					
Age, y	36 (29–42) (n = 133)	−0.02 (−.04 to −.00)^a^	32 (27–38) (n = 133)	−0.01 (−.02 to .01)	.02
Location of residence			136		
Town	44/134 (32.8)	Reference	63/136 (46.3)	Reference	.05
Estate	35/134 (26.1)	−0.09 (−.57 to .40)	20/136 (14.7)	0.45 (.02 to .88)^a^	
SFA	19/134 (14.2)	0.28 (−.30 to .87)	21/136 (15.4)	0.66 (.24 to 1.08)^b^	
RTC	36/134 (26.9)	0.34 (−.12 to .80)	32/136 (23.5)	0.67 (.29 to 1.05)^c^	
Residence duration, y	10 (3–28) (n = 133)	−0.02 (−.03 to −.00)^a^	7 (3–21) (n = 133)	0.01 (−.00 to .02)^d^	.28
Marital history					.19
Previously married	103/134 (76.9)	Reference	106/136 (77.9)	Reference	
Still in union	14/134 (10.5)	0.64 (.06 to 1.22)^a^	7/136 (5.15)	1.11 (.44 to 1.78)^b^	
Never married single	17/134 (12.7)	0.37 (−.22 to .95)	23/136 (16.9)	−0.09 (−.49 to .31)	
Education level					.11
None/primary	57/133 (42.9)	Reference	45/135 (33.3)	Reference	
Secondary/higher	76/133 (57.1)	0.75 (.40 to 1.11)^c^	90/135 (66.7)	0.40 (.07 to .73)^a^	
Occupation					.01
Formal employment	20/132 (15.5)	Reference	8/132 (6.82)	Reference	
Informal employment	43/132 (32.6)	−0.11 (−.74 to .51)	64/132 (48.5)	0.30 (−.36 to .96)	
Unemployed	69/132 (52.3)	0.14 (−.45 to .72)	59/132 (44.7)	0.20 (−.47 to .87)	
Occupation is sex worker	8/132 (6.06)	−0.11 (−.86 to .64)	27/132 (20.5)	0.02 (−.36 to .39)	<.01
Cohabit with a partner	24/134 (17.9)	0.54 (.05 to 1.02)^a^	15/136 (11.0)	−0.38 (−.88 to .12)^d^	.11
Age at sexual debut, y					.22
12–16 (Q1)	38/133 (28.5)	Reference	48/136 (35.3)	Reference	
17–18 (Q2)	39/133 (29.3)	0.12 (−.34 to .58)	46/136 (33.8)	−0.24 (−.61 to .13)	
19–20 (Q3)	30/133 (22.6)	0.49 (−.03 to 1.00)	19/136 (14.0)	0.01 (−.45 to .47)	
≥21 (Q4)	26/133 (19.6)	0.01 (−.52 to .53)	23/136 (16.9)	0.27 (−.17 to .71)	
Age at sex work debut, y					<.01
12–20 (Q1)	25/134 (18.7)	Reference	48/136 (35.3)	Reference^d^	
21–25 (Q2)	25/134 (18.7)	−0.09 (−.68 to .49)	38/136 (27.9)	−0.12 (−.52 to .28)	
26–31 (Q3)	40/134 (29.9)	−0.22 (−.77 to .33)	32/136 (23.5)	−0.12 (−.52 to .29)	
32–54 (Q4)	44/134 (32.8)	−0.37 (−.90 to .17)	18/136 (13.2)	−0.29 (−.79 to .21)	
Age at sex work debut, y	29 (22–33) (n = 134)		23 (20–29) (n = 136)		<.01
Time in sex work, y	5 (2–9) (n = 134)	−0.02 (−.04 to .01)^d^	7 (3–13) (n = 136)	0.00 (−.02 to .03)	.01
Travelled to sell sex					.15
Never	102/134 (76.1)	Reference	96/136 (70.6)	Reference	
>1 mo	12/134 (8.96)	0.04 (−.56 to .65)	23/136 (16.9)	0.44 (.02 to .86)^a^	
In past mo	20/134 (14.9)	−0.57 (−1.10 to −.04)^a^	17/136 (12.5)	0.05 (−.43 to .53)	
STI in last year	19/134 (14.2)	−0.63 (−1.16 to −.10)^a^	19/136 (14.0)	0.12 (−.33 to .58)	.96
Typically expect amount per night of sex	56/134 (41.8)	…	108/136 (79.4)	…	<.01
Typically expect amount per short duration	48/134 (35.8)	…	101/136 (74.3)	…	<.01
Typically expect amount per longer period (weekly, fortnightly, or monthly)	68/134 (50.8)	…	77/136 (56.6)	…	.33
B: Characteristics of the encounter with the most-recent commercial partner					
Price received, US$	10 (5–20) (n = 134)	…	10 (5–20) (n = 136)	…	.48
Place for sex negotiation^e^					.15
Private location	66/134 (49.3)	Reference	64/132 (47.1)	Reference	
Drinking location	17/134 (12.7)	0.12(−.48 to .71)	29/132 (21.3)	−0.24 (−.64 to .15)	
Public location	51/134 (38.1)	0.06 (−.34 to .46)	43/132 (31.6)	0.13 (−.23 to .48)	
Location of the transaction					.85
Urban	75/134 (56.0)	Reference	74/135 (54.8)	Reference	
Rural location	59/134 (44.0)	−0.33 (−.70 to .04)^d^	61/135 (45.2)	−0.06 (−.38 to .26)	
Type of sex partner^f^					<.01
New commercial partner	37/134 (27.6)	Reference	41/132 (30.2)	Reference	
Repeat commercial partner (pays per encounter)	19/134 (14.2)	0.02 (−.52 to .56)	50/132 (36.8)	0.05 (−.31 to .41)	
Commercial partner pays on credit	56/134 (41.8)	−0.15 (−.57 to .28)	31/132 (22.8)	0.43 (.01 to .85)^a^	
Regular commercial partner (gives according to my needs)	22/134 (16.4)	0.05 (.49 to .59)	14/132 (10.3)	−0.10 (−.67 to .46)	
Type of payment^g^					.08
Commodities	28/117 (23.9)	…	19/126 (15.1)	…	
Cash	89/117 (76.1)	…	107/126 (84.9)	…	
Age of sex partner, y					.03
<35	42/129 (32.6)	Reference	63/131 (48.1)	Reference	
35–44	48/129 (37.2)	−0.34 (−.79 to .12)	41/131 (31.3)	0.19 (−.16 to .54)	
≥45	39/129 (30.2)	−0.41 (−.87 to .05)^d^	27/131 (20.6)	0.04 (−.36 to .45)	
Type of encounter					.58
Full night	78/134 (58.2)	Reference	83/135 (61.5)	Reference	
Short duration	56/134 (41.8)	−0.49 (−.89 to −.19)^b^	52/135 (38.5)	−0.29 (−.61 to .03)^d^	
Sex acts, no.	2 (1–3) (n = 133)		2 (2–3) (n = 133)	0.19 (.08 to .31)^b^	.04
Dry sex	29/133 (21.8)	0.25 (−.19 to .70)	35/133 (26.3)	0.55 (.20 to .90)^b^	.39
Condom use					.80
100% (consistent)	84/133 (63.2)	Reference	82/133 (61.7)	Reference	
<100% (inconsistent)	49/133 (36.8)	0.07 (−.32 to .45)	51/133 (38.4)	0.38 (.07 to .70)^a^	
Client asked for condom^h^	73/134 (54.5)	0.12 (−.26 to .49)	74/135 (54.8)	0.48 (.18 to .78)^b^	.99
FSW asked for condom^h^	105/129 (81.4)	−0.05 (−0.47 to .38)	120/133 (90.2)	−0.54 (−1.00 to −.08)^a^	.04

Data are proportion (%) of participants or median value (interquartile range). Differences between FSW1 and FSW2 were calculated using χ^2^ and Fisher exact test, for categorical variables, and Wilcoxon rank sum tests, for continuous variables. Variables that were significant at a *P* value of < .2 were included in the multivariable models.

Abbreviations: CI, confidence interval; RTC, roadside trading center; SFA, subsistence farming area; STI, sexually transmitted infection; ZINB, zero-inflated negative binomial.

^a^ *P* < .05.

^b^ *P* < .01.

^c^ *P* < .001.

^d^ *P* < .2.

^e^ Private locations were FSWs’, clients’, or other people's homes; drinking locations included bars, bottle stores, and nightclubs; public locations included bus stops, roadsides, markets, and church.

^f^ Clients who paid on credit paid a prenegotiated amount at an agreed future point (eg, in a month or fortnight). Short durations typically involve only 1 act of sex, in contrast to a full night, during which there will be several acts of sex.

^g^ Missing zero payments.

^h^ Responses of “don't know” were excluded.

The intraclass cluster coefficient for area was 0.026 (*P* = .07), suggesting no significant evidence of between-area differences.

### Client and FSW Preferences for Protected Sex and Association With Condom use

Most FSWs (83.6%) requested condom use with their most-recent client, but only 54.4% reported their client requested condoms (Table 1). The total number of sex acts and the number of protected acts were correlated when clients requested condom use (Spearman rho = 0.71; *P* < .01) but uncorrelated when they did not (rho = 0.07; *P* = .5). The same patterns were found irrespective of what women requested: when both clients and FSWs requested condoms and when clients but not FSWs requested condom use, total and protected acts were correlated (rho = 0.73 and rho = 0.88, respectively); when neither clients nor FSWs requested condoms and when FSWs but not clients requested condom use, total and unprotected acts were uncorrelated (rho = 0.03 and rho = 0.12, respectively). These results were consistent across both FSW groups (data not shown), although FSW1s were less likely to request condoms (*P* = .04). Condom use did not differ by client type for FSW1s (*P* = .7) or FSW2s (*P* = .09).

### Expected Payments for Sex

Compared with FSW2s, fewer FSW1s reported an expected amount per night or per short duration (Table 1), but similar proportions reported an expected weekly, fortnightly, or monthly payment. There were no significant differences in the distributions of expected payments: median expected amounts for FSW1s and FSW2s were $9.2 (IQR, $5–$15) and $7.5 (IQR, $5–$10), respectively, for short durations (*P* = .4); $30 (IQR, $20–$50) and $30 (IQR, $15–$50), respectively, for longer-term payments (*P* = .9); and $12.5 (IQR, $10–$20) in both groups for a full night.

The median expected amount per full-night encounter among all FSWs was $10.0 (IQR, $10–$20) for protected sex and $20.0 (IQR, $10–$30) for unprotected sex; for a short-duration encounter, the median expected amount was $5.0 (IQR, $5–$10) and $10.0 (IQR, $5–$15) for protected and unprotected sex, respectively (Supplementary Figure 1). Among FSWs who reported charges for both protected and unprotected sex, FSW2s expected higher median payments for unprotected sex versus protected sex ($13.8 [IQR, $10–$25] vs $10.0 [IQR, $7.5–$15]; n = 62; *P* < .01). There was no significant difference in the payments expected for unprotected and protected sex for FSW1s (*P* = .1). FSWs expected higher median amounts for full-night encounters, compared with short-duration encounters ($13.3 [IQR, $10–$23] vs $7.5 [IQR, $5–$10]; *P* < .01). The average expected amount was not significantly different from that received from the most-recent commercial partner (*P* = .3). Cash payments ($10 [IQR, $6.5–$20]) were not significantly different from commodity payments ($10 [IQR, $6–$20]; *P* = .4). Regular clients made up 88% of zero payments (21 of 24).

### Univariable and Multivariable Associations With Price of Sex

Significant determinants in univariable associations of price of sex with most-recent commercial partner that were common to both FSW groups were marital status, cohabitation status, education level, FSW mobility, and number of sex acts (Table 1). Other significant determinants for FSW1s were age, residential duration, recent sexually transmitted infection, duration in sex work, location of most-recent sex, partner's age, and type of encounter. Other significant predictors for FSW2s were area of residence, partner type, dry sex, condom use, and client and FSW requests for condom use.

Table 2 presents the adjusted coefficients for predictors of price in a final model for FSW1s (model 1) and 3 final models for FSW2s (one model involving their most-recent client only [model 2] and two models involving both recent clients, one unclustered [model 3] and one individually clustered [model 4]). In the count part of models 1 and 2, more-educated FSW1s and FSW2s had 73.4% and 40.3% higher average payments, respectively, with their most-recent commercial partners, holding all other variables constant. In models 3 and 4, when both clients were considered, higher education level increased mean payments by 32.8%. The odds of excess zero payments insignificantly decreased with education level for FSW1s but increased for FSW2s. Similarly, the odds of excess zero payments increased with increasing number of sex acts among FSW1s but decreased for FSW2s.

**Table 2.** Multivariable Zero-Inflated Negative Binomial Models Showing the Regression of Price Received for the Most Recent Commercial Sex Act Among Female Sex Workers Reporting 1 Commercial Sex Partner at the Most Recent Sex Act (FSW1) or 2 Commercial Sex Partners at the Most Recent and Second Most Recent Sex Acts (FSW2) in the Past Year Against Its Predictors

Variable	Model 1: FSW1 (n = 127, zeros = 14)		Model 2: FSW2, Most Recent Partner Included (n = 125, zeros = 9)		Model 3: FSW2, Both Partners Included, Unclustered (n = 252, zeros = 18)		Model 4: FSW2, Both Partners Included, Clustered on FSW (n = 252, zeros = 18)	
	Excess Zeros	NB Estimate	Excess Zeros	NB Estimate	Excess Zeros	NB Estimate	Excess Zeros	NB Estimate
	Logit β Coefficient (95% CI)	Log β Coefficient (95% CI)	Logit β Coefficient (95% CI)	Log β Coefficient (95% CI)	Logit β Coefficient (95% CI)	Log β Coefficient (95% CI)	Logit β Coefficient (95% CI)	Log β Coefficient (95% CI)
Marital history (vs previously married)	…	…	Reference	Reference	…	…	…	…
Still in union	…	…	−17.6 (−23 776 to 23 741)	0.99 (.39 to 1.60)^a^	…	…	…	…
Never married	…	…	0.59 (−1.44 to 2.63)	−0.10 (−.48 to .28)	…	…	…	…
Secondary education (vs primary education)	−0.57 (−2.34 to 1.20)	0.55 (.20 to .90)^a^	1.29 (−2.03 to 4.62)	0.36 (.06 to .65)^b^	0.42 (−1.03 to 1.88)	0.28 (.08 to .49)^a^	0.42 (−1.38 to 2.23)	0.28 (.00 to .56)^b^
Years of residence	−0.09 (−.28 to .10)	−0.02 (−.03 to −.00)^b^	…	-…	…	…	…	…
Occupation is sex worker (vs not)	…	…	…	…	−21.24 (−46 010 to 45 967)^c^	0.37 (.12 to .62)^a^	−21.2 (−22.6 to −19.9)^d^	0.37 (.04 to .69)^b^
Most recent sex partner vs new client	…	…	…	…	Reference	Reference	Reference	Reference
Repeat client	…	…	…	…	−1.06 (−3.58 to 1.46)	0.25 (−.01 to .52)	−1.06 (−4.26 to 2.14)	0.25 (−.06 to .57)
Regular client (pay on credit)	…	…	…	…	1.07 (−.87 to 3.00)	0.43 (.15 to .71)^a^	1.07 (−1.17 to 3.31)	0.43 (.05 to .82)^b^
Regular client (pay according to FSW need)	…	…	…	…	−0.47 (−3.40 to 2.46)	−0.23 (−.59 to .13)	−0.47 (−3.74 to 2.80)	−0.23 (−.63 to .17)
Dry sex (vs no dry sex)	…	…	…	…	0.96 (−.50 to 2.43)	0.44 (.20 to .68)^d^	0.96 (−1.15 to 3.08)	0.44 (.10 to .78)^b^
No. of sex acts >1	0.16 (−0.68 to 1.00)	0.27 (.10 to .43)^a^	−0.22 (−.97 to .53)	0.15 (.06 to .25)^a^	−0.26 (−.89 to .36)	0.12 (.03 to .20)^a^	−0.26 (−1.05 to .52)	0.12 (.03 to .21)^a^
Client does not request condom use (vs client requests)	…	…	0.13 (−1.63 to 1.89)	0.45 (.16 to .73)^a^	…	…	…	…
Unprotected sex (vs condom use)	…	…	…	…	1.11 (−.24 to 2.46)	0.36 (.14 to .57)^d^	1.11 (−.56 to 2.78)	0.36 (.09 to .62)^a^
	Value		Value		Value		Value	
Vuong test of ZINB vs standard NB	z = 1.49, *P* = .068		z = 1.51, *P* = .065		z = 2.87, *P* < .01		…	
Log likelihood	−469		−454		−913		−913	
AIC	956.8		935.3		1864.2		1864.2	
BIC	982.4		974.9		1931.3		1931.3	

All models were zero-inflated negative binomial regressions. Missing payments were omitted. Positive coefficients indicate price is positively associated with the predictor, whereas negative coefficients indicate negative correlations.

Abbreviations: AIC, Akaike information criterion; BIC, Bayesian information criterion; CI, confidence interval; NB, negative binomial; ZINB, zero-inflated negative binomial.

^a^ *P* < .01.

^b^ *P* < .05.

^c^ Some cells have zero counts.

^d^ *P* < .001.

For each additional year a FSW1 resides in the area, their expected mean payment decreases by 1.5%, but for each additional sex act, their expected mean payment increases by 30.9%. Marital status and client preference for condom use were both significantly associated with price at the most-recent sex act for FSW2s (model 2), but when both partners of FSW2s were included, only partner type, dry sex, sex work as an occupation, and condom use were significantly associated with price (models 3 and 4).

In the count process of models 3 and 4, holding all other variables constant, unprotected sex increased the mean payment by 42.9%, clients who paid on credit and repeat clients (vs new clients) increased the expected payment by 56.2% and 28.3%, respectively. Each additional sex act increased the mean payment by 12.6%; providing dry sex and reporting their occupation as sex work increased the mean payment by 56.9% and 44.4%, respectively. The odds of excess zero payments significantly decreased when sex work was reported as an occupation but insignificantly decreased for repeat clients (vs new clients) and with additional sex acts independently (model 4). There was a nonsignificant increase in the odds of excess zero payments with dry sex, regular clients, and unprotected sex independently.

In sensitivity analyses, inclusion of missing values did not qualitatively alter findings (Supplementary Materials).

## DISCUSSION

This study improves understanding of how payments for commercial encounters are determined and what factors are important to price negotiations. The median price for the most-recent commercial sex per night ($10 [IQR, $5–$20]) was consistent with the range of prices (2010 real value, US$11.6–$17.1) in studies conducted prior to the economic crisis elsewhere in Zimbabwe [14, 24]. Short-duration encounters ($10 [IQR, $5–15]) were slightly higher than per-act prices in other studies (2010 US$2.5–$7.5) [25], which likely reflects short durations occasionally consisting of >1 act. Most payments were in cash, but commodity payments were of equivalent value.

Few studies have explored rural sex work in sub-Saharan Africa and typically only in comparison with urban FSWs [26, 27]. This in-depth study of rural FSWs identified distinct operations: FSW1s tended to sell sex on credit to regular clients who paid weekly or monthly, and FSW2s regularly sought both pay-per-encounter and on-credit clients, with the first forming the majority of recent clients. Despite these differences, the price for the most-recent sex encounter was comparable between FSW1s and FSW2s, and condom use (63.2% and 61.7%, respectively) was similar to levels reported by FSWs in a nearby city (65.2%) [28].

In a collapsed economy, with a reduced demand for paid sex [10, 29], extreme poverty might have reduced differences in prices between protected and unprotected sex. However, we found that, within a work environment in which clients dominated decisions over safer sex, the more-professional FSWs, who were more experienced at selling sex, effectively negotiated the terms of the transaction: unprotected sex increased the mean payment by 43%, compared with protected sex, confirming initial findings. In contrast, prices for protected and unprotected sex were not statistically different among less-professional FSWs, perhaps reflecting limited capacity to demand higher prices, particularly with regular clients with whom issues of trust, rather than price, may determine unprotected sex [13]. Meanwhile, FSW2s also provided dry sex for higher prices. Dry sex weakened but did not eliminate the condom-use effect, suggesting interference but not deterrence of condom use [30].

In multivariable analyses, education level and number of sex acts were consistently positively associated with price across FSWs and partners. Although greater education level is often viewed as a means to increase job opportunities, reduce reliance on commercial sex, or exit sex work, it also benefits FSWs remaining in sex work, who obtain higher prices. Numeracy and literacy may help with negotiating prices [4], and they are also linked with higher-paid clients [18].

Repeat and regular clients paid higher prices than new clients with FSW2s, contrasting with research in US sex workers who gave repeat clients discounts [16] but consistent with Kenyan FSWs receiving higher payments from regular partners [27]. Average payments reflect the count process and not the total population effect, which is influenced by the excess zeros. Higher prices from regular clients might reflect selection bias: wealthier clients (who pay more for sex) can afford to buy sex regularly [29]; FSWs might select wealthier clients as regular partners.

In contrast to studies elsewhere, we found no evidence of associations of price with age [27], duration in sex work [3], location or place where sex was negotiated [1, 2, 26], or encounter type [16]. Absence of an association with age might reflect the maturity of the FSW population and underrepresentation of FSWs aged <18 years and women starting sex work. Other significant variables likely capture some of these effects: for example, residential duration was negatively associated with price and was positively associated with age (data not shown), and numbers of acts were higher in full-night encounters (*P* < .01). Levels of consistent condom use comparable to those in other studies [28] suggest that the informal confidential voting interviews minimized social-desirability bias.

Despite enthusiasm for biomedical interventions, our results demonstrate potential gains from condom promotion: strengthening preferences for protected sex, including for less-visible FSWs, could make unprotected sex less affordable [4]. Such strategies would benefit from enhanced social capital and collective action (eg, fixing prices) to reduce competitive pressure for unprotected sex [13].

This is the first analysis of the determinants of payments for sex among FSWs in Zimbabwe. Within a context where clients' preferences governed the use of condoms, professional FSWs negotiated higher prices for unprotected sex. High individual capital among hidden and professional FSWs suggests that strengthening preferences for condom use could be effective in maintaining high levels of safer sex.

## Supplementary Data

Supplementary materials are available at *The Journal of Infectious Diseases* online (http://jid.oxfordjournals.org). Supplementary materials consist of data provided by the author that are published to benefit the reader. The posted materials are not copyedited. The contents of all supplementary data are the sole responsibility of the authors. Questions or messages regarding errors should be addressed to the author.

## References

[1] Johnston CL, Callon C, Li K, Wood E, Kerr T (2010). Offer of financial incentives for unprotected sex in the context of sex work. Drug Alcohol Rev.

[2] Ntumbanzondo M, Dubrow R, Niccolai LM, Mwandagalirwa K, Merson MH (2006). Unprotected intercourse for extra money among commercial sex workers in Kinshasa, Democratic Republic of Congo. AIDS Care.

[3] Rao V, Guptab I, Lokshina M, Smarajit Jana S (2003). Sex workers and the cost of safe sex: the compensating differential for condom use among Calcutta Prostitutes. J Dev Econ.

[4] Gertler P, Shah M, Bertozzi SM (2005). Risky business: the market for unprotected commercial sex. J Polit Econ.

[5] Dupas P, Robinson J (2012). The (hidden) costs of political instability: Evidence from Kenya's 2007 election crisis. J Dev Econ.

[6] Robinson J, Yeh E (2011). Transactional sex as a response to risk in Western Kenya. Am Econ J Appl Econ.

[7] Schur N, Mylne A, Mushati P (2013). The effects of changes in household wealth on HIV risk in Manicaland, Zimbabwe [oral presentation].

[8] Gregson S, Gonese E, Hallett TB (2010). HIV decline in Zimbabwe due to reductions in risky sex? Evidence from a comprehensive epidemiological review. Int J Epidemiol.

[9] Halperin DT, Mugurungi O, Hallett TB (2011). A surprising prevention success: why did the HIV epidemic decline in Zimbabwe?. PLOS Med.

[10] Elmes J, Nhongo K, Skovdal M (2013). PCS1.03: The changing nature and contribution of sex work to HIV transmission during the economic crisis in Zimbabwe [oral presentation].

[11] Baird S, Chirwa E, McIntosh C, Ozler B (2010). The short-term impacts of a schooling conditional cash transfer program on the sexual behavior of young women. Health Econ.

[12] Gregson S, Mushati P, White PJ, Mlilo M, Mundandi C, Nyamukapa C (2004). Informal confidential voting interview methods and temporal changes in reported sexual risk behaviour for HIV transmission in sub-Saharan Africa. Sex Transm Infect.

[13] Wojcicki JM, Malala J (2001). Condom use, power and HIV/AIDS risk: sex-workers bargain for survival in Hillbrow/Joubert Park/Berea, Johannesburg. Soc Sci Med.

[14] Wilson D, Chiroro P, Lavelle S, Mutero C (1989). Sex worker, client sex behaviour and condom use in Harare, Zimbabwe. AIDS Care.

[15] de la Torre A, Havenner A, Adams K, Ng J (2010). Premium sex: Factors influencing the negotiated price of unprotected sex by female sex workers in Mexico. J Appl Econ.

[16] Levitt S, Venkatesh S An Empirical Analysis of Street-Level Prostitution.

[17] Deering KN, Boily MC, Lowndes CM (2011). A dose-response relationship between exposure to a large-scale HIV preventive intervention and consistent condom use with different sexual partners of female sex workers in southern India. Bmc Public Health.

[18] Lopman B, Lewis J, Nyamukapa C (2007). HIV incidence and poverty in Manicaland, Zimbabwe: is HIV becoming a disease of the poor?. AIDS.

[19] Snijders TAB, Bosker RJ (2012). Multilevel analysis : an introduction to basic and advanced multilevel modeling.

[20] Hilbe JM (2011). Negative binomial regression.

[21] Rothman KJ, Greenland S, Lash TL (2008). Modern epidemiology.

[22] Long JS, Freese J (2006). Regression models for categorical dependent variables using Stata.

[23] Zuur AF (2009). Mixed effects models and extensions in ecology with R.

[24] Ray S, van de Wijgert J, Mason P, Ndowa F, Maposhere C (2001). Constraints faced by sex workers in use of female and male condoms for safer sex in urban Zimbabwe. J Urban Health.

[25] Cowan FM, Hargrove JW, Langhaug LF (2005). The appropriateness of core group interventions using presumptive periodic treatment among rural Zimbabwean women who exchange sex for gifts or money. J Acquir Immune Defic Syndr.

[26] Pickering H, Quigley M, Hayes RJ, Todd J, Wilkins A (1993). Determinants of condom use in 24000 prostitute client contacts in the Gambia. AIDS.

[27] Voeten H, Egesah OB, Varkevisser CM, Habbema JDF (2007). Female sex workers and unsafe sex in urban and rural Nyanza, Kenya: regular partners may contribute more to HIV transmission than clients. Trop Med Int Health.

[28] Cowan FM, Mtetwa S, Davey C (2013). Engagement with HIV prevention treatment and care among female sex workers in Zimbabwe: a respondent driven sampling survey. PLoS One.

[29] Muchini B, Benedikt C, Gregson S (2011). Local perceptions of the forms, timing and causes of behavior change in response to the AIDS epidemic in Zimbabwe. AIDS Behav.

[30] Civic D, Wilson D (1996). Dry sex in Zimbabwe and implications for condom use. Soc Sci Med.

